# Single molecule, near full-length genome sequencing of dengue virus

**DOI:** 10.1038/s41598-020-75374-1

**Published:** 2020-10-23

**Authors:** Thiruni N. Adikari, Nasir Riaz, Chathurani Sigera, Preston Leung, Braulio M. Valencia, Kirston Barton, Martin A. Smith, Rowena A. Bull, Hui Li, Fabio Luciani, Praveen Weeratunga, Tun-Linn Thein, Vanessa W. X. Lim, Yee-Sin Leo, Senaka Rajapakse, Katja Fink, Andrew R. Lloyd, Deepika Fernando, Chaturaka Rodrigo

**Affiliations:** 1 School of Medical Sciences, University of New South Wales, Sydney, Australia; 2 Institute for Combinatorial Advanced Research and Education, Sir John Kotelawala Defence University, Ratmalana, Sri Lanka; 3 Kirby Institute, University of New South Wales, Sydney, Australia; 4 Department of Microbiology, Hazara University, Mansehra, KPK Pakistan; 5 Department of Parasitology, Faculty of Medicine, University of Colombo, Colombo, Sri Lanka; 6 Garvan Institute of Medical Research, Sydney, Australia and St-Vincent’s Clinical School, Faculty of Medicine, UNSW, Sydney, Australia; 7 CHU Sainte-Justine Research Centre, Montreal, Canada; 8 Department of Biochemistry and Molecular Medicine, Université de Montréal, Montreal, Canada; 9 Department of Clinical Medicine, Faculty of Medicine, University of Colombo, Colombo, Sri Lanka; 10 National Centre for Infectious Diseases, Singapore, Singapore; 11 Department of Infectious Diseases, Tan Tock Seng Hospital, Singapore, Singapore; 12 Department of Medicine, Yong Loo Lin School of Medicine, National University of Singapore, Singapore, Singapore; 13 Lee Kong Chian School of Medicine, Singapore, Singapore; 14 Agency for Science, Technology and Research, Singapore, Singapore

**Keywords:** Next-generation sequencing, RNA sequencing, Genome informatics

## Abstract

Current methods for dengue virus (DENV) genome amplification, amplify parts of the genome in at least 5 overlapping segments and then combine the output to characterize a full genome. This process is laborious, costly and requires at least 10 primers per serotype, thus increasing the likelihood of PCR bias. We introduce an assay to amplify near full-length dengue virus genomes as intact molecules, sequence these amplicons with third generation “nanopore” technology without fragmenting and use the sequence data to differentiate within-host viral variants with a bioinformatics tool (Nano-Q). The new assay successfully generated near full-length amplicons from DENV serotypes 1, 2 and 3 samples which were sequenced with nanopore technology. Consensus DENV sequences generated by nanopore sequencing had over 99.5% pairwise sequence similarity to Illumina generated counterparts provided the coverage was > 100 with both platforms. Maximum likelihood phylogenetic trees generated from nanopore consensus sequences were able to reproduce the exact trees made from Illumina sequencing with a conservative 99% bootstrapping threshold (after 1000 replicates and 10% burn-in). Pairwise genetic distances of within host variants identified from the Nano-Q tool were less than that of between host variants, thus enabling the phylogenetic segregation of variants from the same host.

## Introduction

Dengue is a major public health problem with nearly half the global population at risk of infection^1,2^. The incidence of dengue has increased by more than 30-fold in the last 50 years, and the disease is now endemic in approximately 125 countries^3^. DENV is a positive sense single stranded RNA virus belonging to the genus *Flavivirus* in the family *Flaviviridae*. The open reading frame (ORF) of the virus consists of seven non-structural (NS1, NS2A, NS2B, NS3, NS4A, NS4B, NS5) and 3 structural proteins (Capsid, Precursor membrane protein, Envelope) with an overall genome size of approximately 10.7 kb (3391 amino acids)^4^. DENV is further divided in to four serotypes (DENV 1–4) that share up to 75% sequence similarity between them. Each serotype is further subdivided into genotypes, which present up to 94% sequence similarity^5^.

Clinical manifestations of dengue vary from asymptomatic infection to simple dengue fever to more severe and potentially fatal dengue shock syndrome^6^. Research on the determinants contributing to disease severity mostly focuses on host immunology. For instance, non-neutralizing but cross-reactive immunity induced by primary infection is a recognized risk factor for the development of severe dengue during a second infection^7^. Virological markers of disease severity are less studied in dengue. In other RNA virus infections such as HIV and hepatitis C virus (HCV) infections, virological factors (epistasis, founder variants, genomic variability) are known to correlate with clinical outcomes and disease severity^8–10^. Epidemiologically, the frequency of severe dengue cases correlates with exposure to new DENV serotypes, which links viral genomics to dengue epidemics^11^. Hence, maintaining virological surveillance via sequencing of circulating variants can be helpful to illustrate and predict new epidemics and their severity. However, genomic sequencing requires setting up infrastructure for sequencing facilities—a costly exercise beyond the economical capabilities of many low- and middle- income countries where DENV is predominantly endemic.

Another barrier to study viral genomics in dengue is the difficulty in generating unbiased full-length DENV genomes and successfully sequencing them as intact genomes. The current gold standard employs PCR and capillary sequencing to characterize the DENV genome in at least five overlapping amplicons^12^. This is how almost all full-length DENV genomes in public databases have been sequenced thus far. However, this process is labour intensive, costly and imposes limitations for studies that require identification of true within-host variants (e.g. for the estimation of founder variants and epistatic interactions of the DENV genome). Alternatively, state-of-the-art next generation sequencing methods, such as paired-end short read sequencing on the Illumina platform, enables detailed characterization of low frequency within-host single nucleotide polymorphisms (SNPs) of RNA viruses. However, the short read (75–300 nt) read output by this platform needs to be concatenated using statistical algorithms (haplotype reconstruction) if within host variants are to be reconstructed to understand which two or more SNPs co-occur in the same genome. Unfortunately, there is limited agreement among the multiple statistical algorithms that are available for use when generating full length “true variants”^13,14^. Therefore, it is currently not possible to accurately identify co-occurring SNPs within the same variant, if they are separated by more than 600–1000 bps across the genome. This places a limitation on in-depth virological analyses predicated on genetic data. Whole genome amplification and sequencing (of intact full-length amplicons) presents an alternative approach to bypass haplotype reconstruction. Doing so in a cost-effective and high throughput manner will improve the utility of a pipeline designed for this purpose.

Oxford nanopore technologies (ONT), a third-generation sequencing technology, can overcome this problem through two distinct advantages; cost effectiveness and the capacity to generate long reads (> 2 MB nts) that far exceed the average length of many RNA viruses, including dengue. The MinION sequencer is a hand-held portable device that requires fewer resources than other mainstream NGS platforms, such as paired-end short read technology generated by Illumina and single molecule real-time sequencing generated by PacBio^15^. The MinION has been applied in RNA virus sequencing during epidemics with success in remote locations without sophisticated laboratory support^16,17^.

This paper introduces a novel assay capable of generating near full-length DENV genomes as complete molecules. These are then sequenced intact on the nanopore platform, and the consensus sequence output is assessed for accuracy and cost effectiveness using the paired- end short read technology as the comparator. Furthermore, a novel bioinformatics tool is utilized for differentiating within-host DENV variants and their relative frequencies from the nanopore sequencing output.

## Results

### Generation of near full-length amplicons

**Figure 1:**
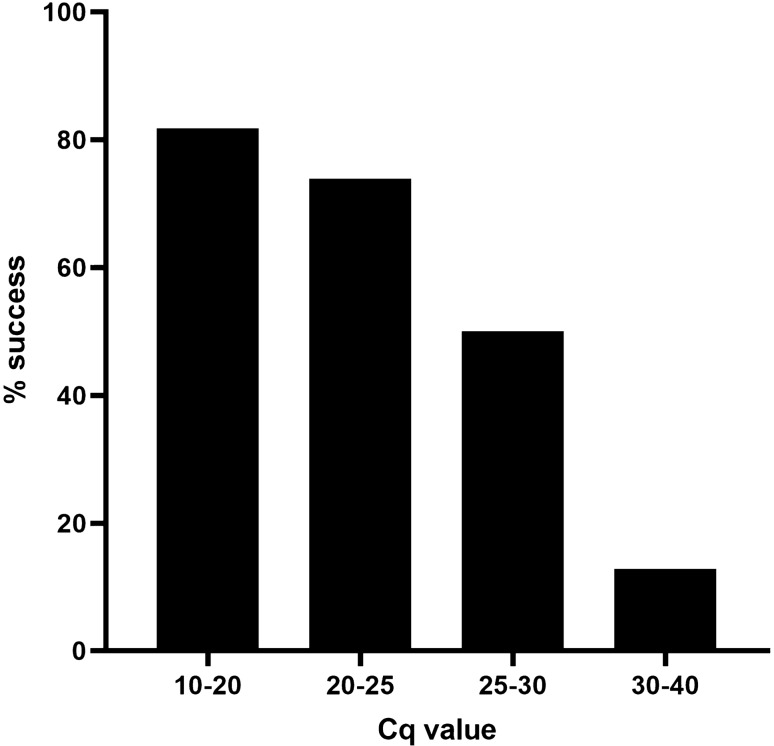
Success rate of near-full genome amplification assay stratified by the Cq value of the quantitative PCR per sample. Please see Supplementary Tables 1 and 2 for corresponding viral loads.

The new assay developed by us which is detailed in supplementary methods was tested on one hundred and four clinical samples (DENV1–18, DENV2–75, DENV3–7, DENV4–4). Near full-length amplicons were visible on gel electrophoresis in 47 samples and corresponding genomes were identified from nanopore sequencing in 45 of these. An additional 6 sequences were recovered by the more sensitive Illumina sequencing from the samples where nanopore sequencing was unsuccessful (see below), indicating successful amplicon generation in 51 samples (DENV1–7, DENV2–41, DENV3–3). The Cq values obtained from qPCR, which are inversely related to the initial viral load of the sample, ranged from 13.45 to 40.52 for all 104 samples (Supplementary Table 1). The successful samples had a significantly lower mean Cq value compared to others (24.17, SD ± 4.8 vs. 31.24, SD ± 6.0, *p* < 0.001). When divided in to quartiles based on the Cq value, samples with a higher viral load were more likely to give a positive result compared to those with a lower viral load (82%, 9/11 for Cq values of 10–20 vs. 13%, 5/39 for Cq values of 30–40) (Fig. 1 and Supplementary Table 2). The assay was successful in making amplicons for serotypes 1–3 but not for serotype 4. However, all serotype 4 samples had a Cq value above 36.67. A logistic regression analysis for successful amplicon generation demonstrated that a higher Cq value is significantly associated with a negative result (Adjusted OR: 1.25, 95% CI: 1.13–1.38, *p* < 0.0001) while the serotype did not influence this outcome (*p* > 0.05).

### Coverage and cost effectiveness

All successfully amplified samples were sequenced with Oxford nanopore technology, a third-generation, long read sequencing platform. Samples were multiplexed in a single flow cell for sequencing with either ligation based, or PCR based barcodes for cheaper sequencing. The median number of near-full-length nanopore reads per sample was 959 (Interquartile range: 35–18,939) in successfully sequenced samples. There was a difference in coverage depending on the barcoding method used in a single sequence run. PCR barcoding which allows pooling of a maximum of 96 samples had a lower overall coverage per sample (median: 276, IQR: 32–1295) compared to barcoding by ligation which allows multiplexing of up to 12 samples per run (median: 13,730, IQR: 59–26,914). Nanopore sequencing with PCR barcoding was less expensive than ligation-based barcoding when assuming that the maximum possible number of samples pooled on a single flowcell (AUD 28 vs. 142 per sample, respectively). This estimate is based on commercial sequencing rates in Australia in November 2019 including pre-preparation costs, the cost of a GridION flow cell, and taxes. The comparable cost for Illumina sequencing per sample (with pooling of 96 samples per run) was approximately AUD 100.

### Impact of size selection on the number of near full-length sequences

In a separate experiment, four samples following amplicon generation were subjected to PCR based barcoding with and without size selection simultaneously in two batches. Size selection by gel electrophoresis picks the amplicons at 10 kb band thus increasing the yield of full-length genomes but may also decrease the total number of full-length reads due to loss of sample from additional processing steps. Both batches were sequenced with nanopore technology and results are shown in Table 1. The output displayed high variability from sample to sample, yet the total yield was higher in non-size selected samples versus size selected samples (4–88 times higher) consistently. Reads that approximated the length of the DENV genome were also more prevalent in all the non-size selected samples (1.2–2200 times higher). When these reads (8500–12,000 bp in length) were matched against autologous reference sequences, the total number of mapped reads were again higher with the non-size selected samples.

**Table 1:** Comparison of yield of nanopore reads in the length of interest with and without size selection.

Sample	10 kb band on Gel electrophoresis	No size selection				With size selection			
		Total reads	Reads of 8500–12,000 bps	Matching reads (to autologous reference)	Matching reads as a % of total reads	Total reads	Reads of 8500–12,000 bps	Matching reads (to autologous reference)	Matching reads as a % of total reads
S3	No	463,603	198	192	0.041	116,381	1	1	0.001
S5	Yes	319,676	4417	4297	1.344	3624	2	0	0.000
S24	Yes	1,349,105	13,070	12,808	0.949	301,975	11,021	10,811	3.580
S25	Yes	721,449	76,145	74,694	10.353	43,707	200	196	0.448

### Accuracy of consensus sequences

The accuracy of nanopore sequencing was compared against paired-end short read sequencing by generating consensus sequences per alignment from Illumina and nanopore sequences. On average, the quality score per base position in single reads were much lower with nanopore sequencing compared to Illumina sequencing (On average, Q 7–9 vs. Q 30–40; the Q score indicates the probability of error per base call in a log scale with higher scores indicative of lower probability of an error). All 45 samples sequenced with ONT were re-sequenced with Illumina, as well as an additional 27 samples that were unsuccessful with ONT. Thirty-three samples were successfully sequenced by both platforms. The percent pairwise similarity of Illumina and nanopore generated consensuses was high across these samples (average: 99.7% SD ± 0.34) (Supplementary Table 3). Six samples that failed with nanopore sequencing were successfully sequenced with Illumina even when the gel electrophoresis displayed no band.

### Accuracy of ONT sequencing for phylogenetic analysis

**Figure 2:**
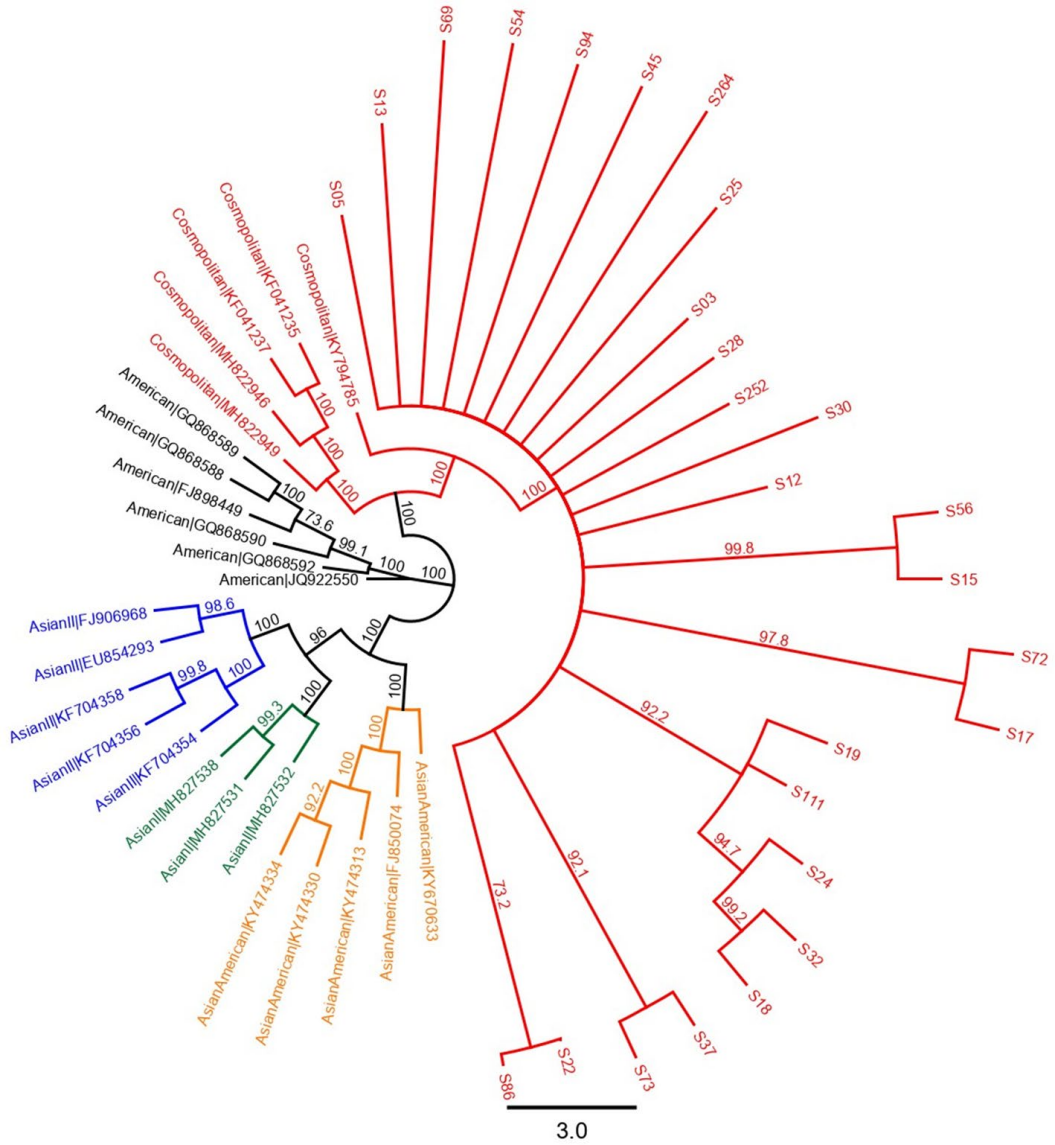
Maximum likelihood phylogenetic tree (consensus of 1000 bootstrap replicates with 99% bootstrap support) showing clustering of DENV2 sequences of this study (red) with Cosmopolitan genotype. Caption: Each color indicates a different genotype of DENV2; Orange: cosmopolitan, Green: American, Blue: Asian American, Pink: Asian I, Black: Asian II.

**Figure 3:**
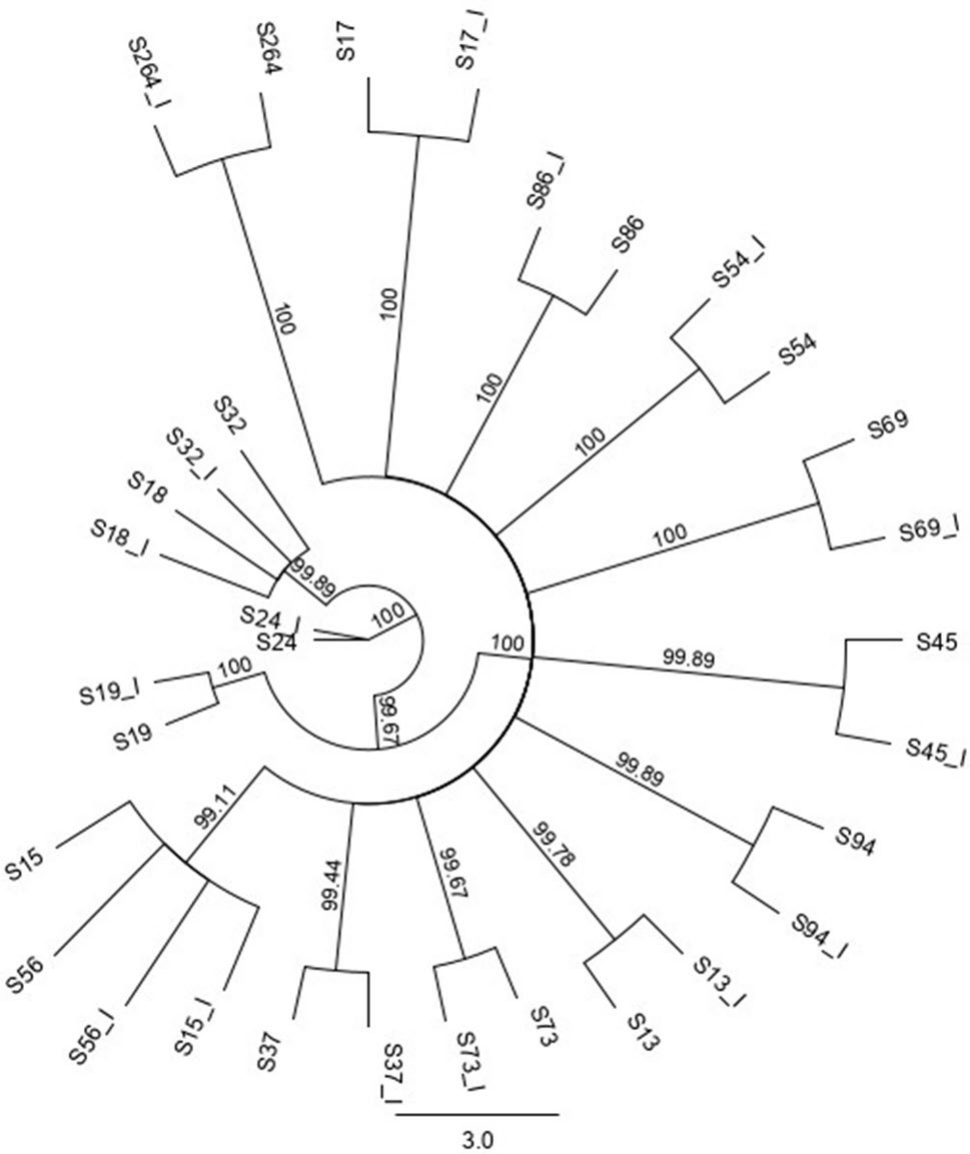
Combined maximum likelihood (consensus of 1000 bootstrap replicates with 99% bootstrap support) of Illumina and nanopore consensuses demonstrating the clustering of each nanopore consensus with its Illumina counterpart (denoted with a _I against the sample number).

**Figure 4:**
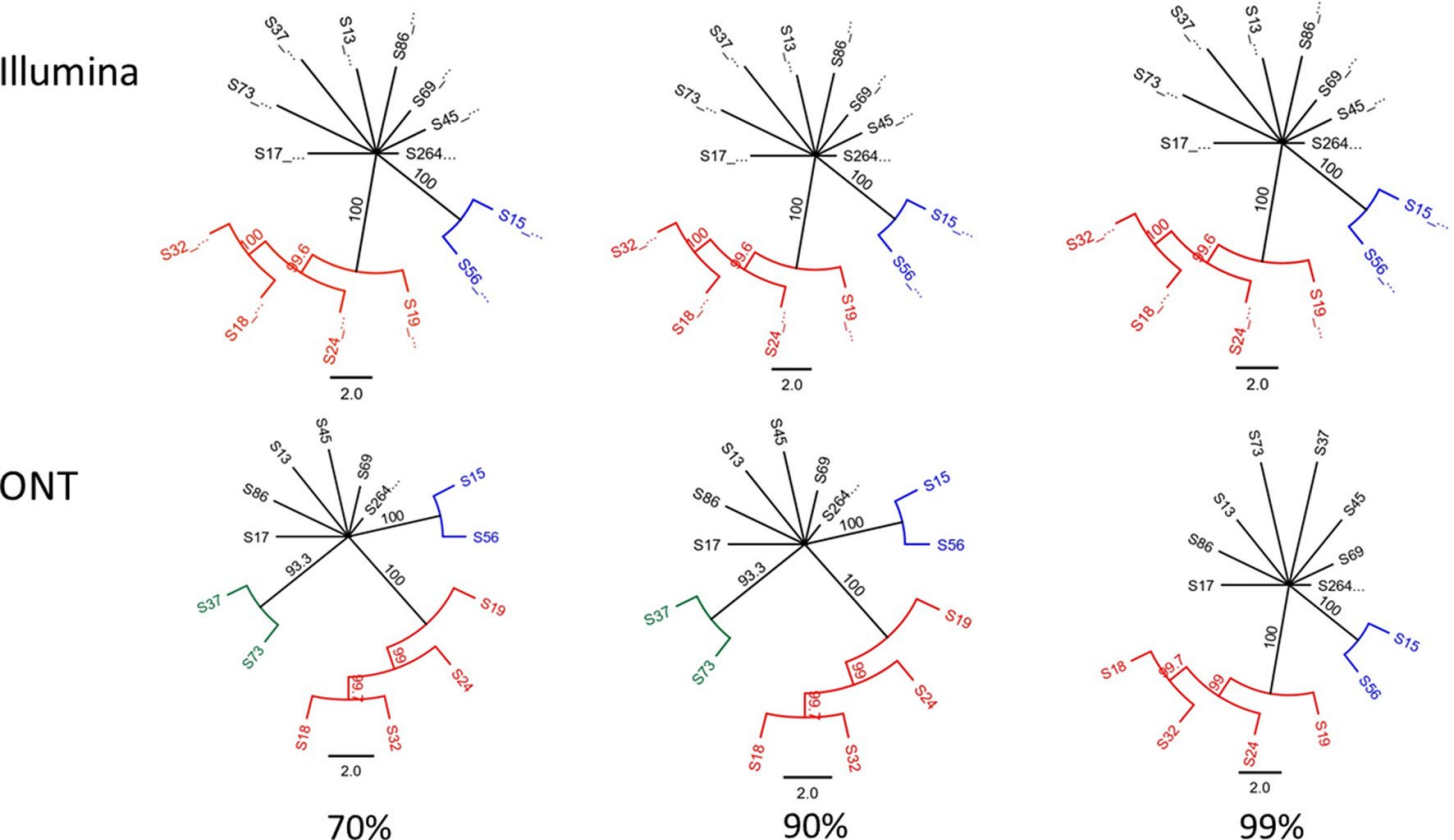
Comparison of Illumina and ONT generated consensuses each with a > 100 coverage throughout the genome with a maximum likelihood phylogenetic tree.

There were 26 DENV2 genomes sequenced on both Illumina and nanopore platforms. These were isolated within a 12-month time period from within the Colombo district, Western Province of Sri Lanka, presumably from the same epidemic. Alignment with publicly available, genotype specific, DENV2 genomes demonstrated that all these sequences were of Cosmopolitan genotype (Fig. 2). A subset of 14 sequences that had > 100 read coverage were used for further comparisons. Two alignments, each made from 14 nanopore and Illumina sequenced genomes, demonstrated comparable pairwise genetic differences within the alignment regardless of the sequencing platform; average of 42.82 (SD ± 17.37) per 10.1 kb genome length for nanopore alignment and 43.72 (SD ± 17.5) for Illumina alignment (for the same genome length). When the sequences were combined in a single phylogenetic tree, all nanopore consensuses paired with their Illumina counterparts (at a bootstrap threshold of 99%) again indicating the comparable effective accuracy across the two sequencing platforms (Fig. 3). When the phylogenetics analysis was repeated for consensuses generated by each sequencing platform separately and by varying the bootstrap threshold (to 70%, 90% and 99%), nanopore consensuses identified an additional cluster with 2–3 sequences (from a total of 3 clusters) at both 70% and 90% thresholds. Identical clusters were observed across Illumina and nanopore trees at a strict bootstrap cut-off of 99% (Fig. 4). After validating the comparable accuracy at a 99% bootstrap threshold across both platforms, another sensitivity analysis was performed to determine if subgenomic regions used for phylogenetics could reproduce the clusters observed with near full-length genome nanopore sequences. Extracted nanopore alignments of Capsid-PrM, Envelope, NS1, NS2, NS3, NS4, NS5 regions (length 793–2700) were used to generate maximum likelihood phylogenetic trees with a bootstrap support of 99%. Only NS1, NS2 and NS4 regions reproduced the two clusters identified from the near full-length genome tree. Although the NS2 region accurately reproduced two clusters, the sub-clusters within one large cluster (of 4 sequences) could not be reproduced (Supplementary Fig. 1). Both NS1 and NS4 regions identified an additional “false” cluster that was not present in the near full-length genome tree which had already been validated against the Illumina generated tree.

When the bootstrap support is set at 70% and 90% nanopore consensus tree identifies an additional cluster not seen with all the Illumina consensus trees. Illumina tree remains consistent regardless of the bootstrap cut-off and agrees with its nanopore counterpart only when the latter has a very conservative 99% bootstrap support for its nodes.

### Characterization of within host variants

**Figure 5:**
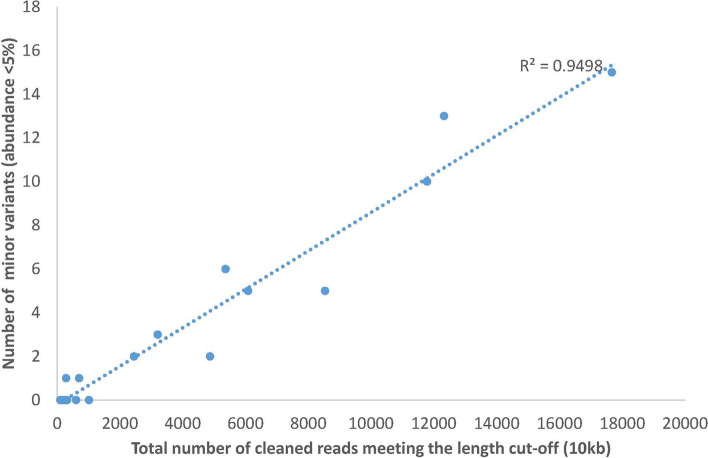
The number of minor variants (< 5% of abundance) reconstructed with Nano-Q tool changes linearly with the total number of eligible reads for the pipeline. However, as most minor variants have an abundance < 1%, detection of more variants (if more reads were available), is unlikely to impact the frequency estimation of major variants.

**Figure 6:**
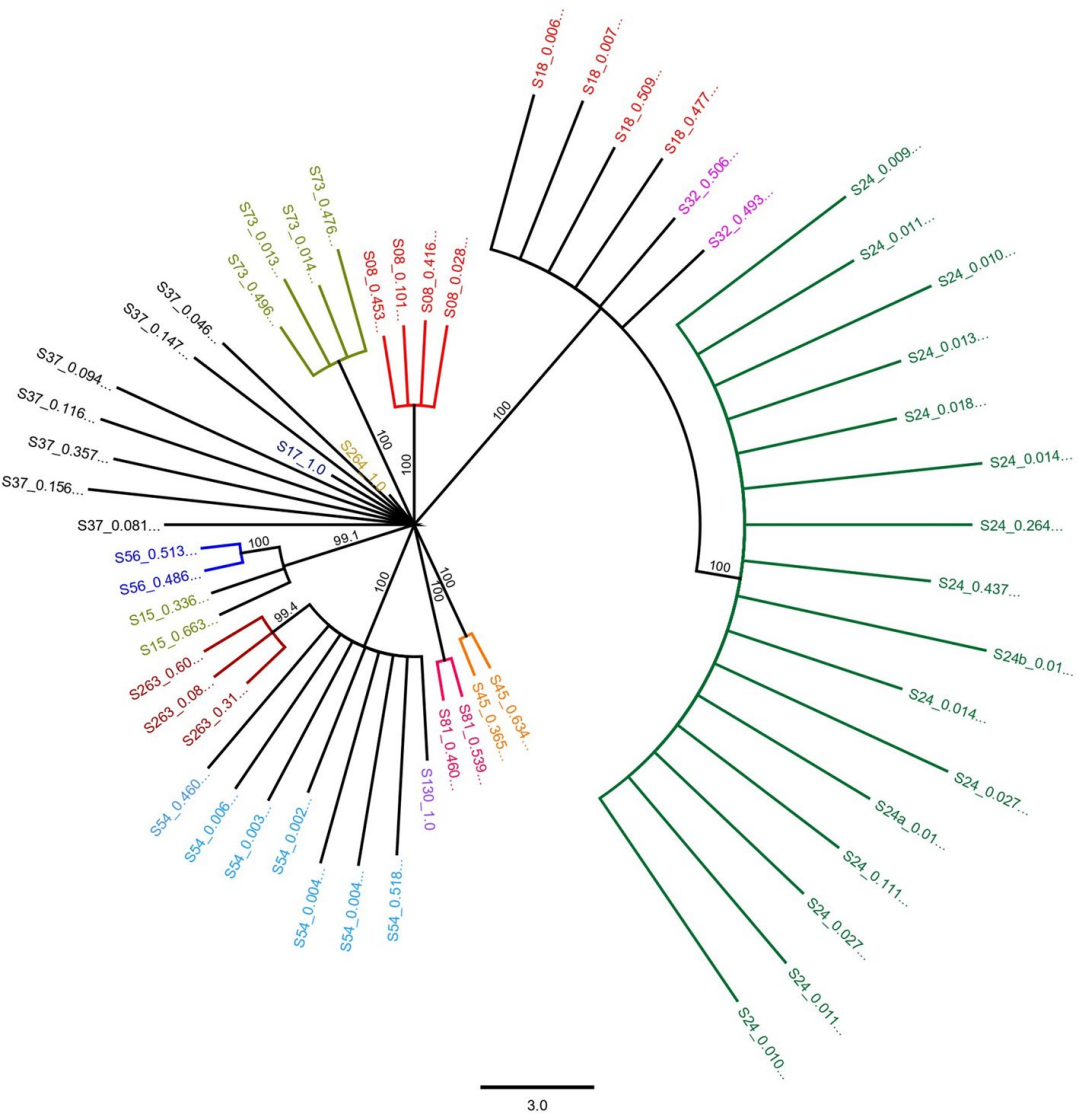
Maximum likelihood phylogenetic tree (consensus of 1000 bootstrap replicates with 99% bootstrap support) of 57 near-full genome within host variants isolated from 14 subjects with DENV2 infection demonstrating clustering of variants by the host. Variants from each host is in the same color. In three subjects, only one variant was found. The relative abundance of the variant is given as a percentage next to variant name (e.g. 0.46 refers to a 46% abundance).

All 45 samples sequenced with nanopore technology were analyzed with Nano-Q tool, an in-house developed bioinformatics tool to characterize within-host RNA virus variants from nanopore sequencing output. The parameters used for the tool and a brief description of each parameter is given in the Supplementary Methods. Of the 45 samples, only 21 samples (DENV1:5, DENV2:14, DENV3:1) had more than 100 reads (range 105–17,672) greater than 10 kb after the initial cleaning steps of the tool, which was established as a lower limit threshold to proceed. The number of within host variants detected ranged from 2 to 27 per sample with mostly 2–3 dominant variants and the rest being minor variants (< 5% abundance). The number of minor variants identified was influenced by the total number of cleaned reads per sample (Fig. 5). The mean maximum pairwise differences of within-host variants (111 variants across 20 subjects) was 7.22 (SD ± 6.54) per approximately 9700 bp (which was the modal length of reconstructed variants). This was less than the between-host consensus sequence variation as shown in the previous section. A maximum likelihood phylogenetic tree of within host variants (99% bootstrap support) for both DENV1 and 2 demonstrated separation of variant clusters by subjects on most of the occasions. For DENV2, out of 14 subjects (57 variants), variants of 10 subjects stood separated from the rest as individual clusters. Of the remaining four subjects, two had only one variant and the variants from the other two subjects were observed in a single cluster (Fig. 6). In five subjects with DENV1 infection which had 51 variants between them, variants of one subject were clearly separated from the rest whereas two more clusters had variants from two subjects in each of them.

## Discussion

This study, provides an original workflow for extracting and amplifying the dengue genome in its entirety, sequencing it as an intact molecule and characterizing within-host variants. It also establishes parameters for accurate phylogenetic interpretation of dengue nanopore sequence data using state-of-the-art, paired-end short read sequencing (Illumina) as a benchmark.

The near-full genome amplification assay described herein successfully amplified sequences from dengue serotypes 1–3 but this was partially influenced by the initial viral load as samples with a low viral load were significantly more likely to yield a (false) “negative” result. We were unable to test dengue serotype 4 as the only four samples available had very low viral loads. Given that the primers were designed by scrutinizing conserved 3′ and 5′ UTR regions of the DENV4 sequences isolated from multiple countries in all endemic continents since 2005, and that the efficacy of the assay did not depend on the serotype for DENV1–3, it is likely that this assay may amplify DENV4 sequences from samples with a higher viral load.

At the consensus level, nanopore generated sequences had more than 99.5% similarity to paired-end short read sequencing data when the coverage per base position exceeded 100. However, the recommended amount of input DNA for nanopore sequencing is much higher than for Illumina sequencing (1000 ng vs. 0.2 ng)^18^ and as demonstrated in this study, highly sensitive Illumina sequencing can produce conclusive data even when gel electrophoresis is negative and when nanopore sequencing produced no reads. Yet, short read sequencing has the inherent weakness of fragmenting the full-length amplicons during library preparation which makes it difficult to characterize within host variants. The quality score per base in single reads was lower in nanopore sequencing compared to Illumina sequencing which generates reads with a 10–100 times better accuracy. However, as shown in this study, the error rate for nanopore sequencing can be compensated by depth of coverage to provide a comparable consensus sequence.

Apart from the technical value of generating full length reads, nanopore sequencing is also attractive for field work and cost-effectiveness given the portability of the hand-held MinION^15^. The assay described in this paper still requires an RNA extraction and a lengthy PCR. However, it removes the need for a sequencing facility with bulky and expensive sequencers that are out of reach in many low- and middle-income countries (including Sri Lanka) where researchers usually send samples to another country to be sequenced. The comparative cost effectiveness benchmarked here against short read (Illumina) sequencing depends on the barcoding method used. PCR based barcoding is significantly cheaper and needs less input DNA but in our initial experiments had a low coverage per sample while the considerably expensive and laborious ligation barcoding had an in-depth coverage. However, our protocol included two size selection steps by gel extraction immediately before and after PCR barcoding, based on the assumption that size selection will increase the yield of full genome reads while filtering out shorter reads. This was based on a previous finding by the authors that short read technologies preferentially sequence shorter input fragments, which was resolved with size selection^19^. However, when this assumption was tested for nanopore sequencing, size selection was shown to be detrimental. This is possibly due to the loss of input DNA and the higher input requirement for nanopore sequencing. Therefore, for PCR barcoding, if samples are multiplexed, size selection is not recommended.

Despite the highly comparable consensus sequences (> 99.5% pairwise similarity) generated across the two sequencing platforms, if short read technology is assumed to be the gold standard, even a 0.5% error rate (50 nucleotides per 10,000 bp of DENV genome) with nanopore consensus may eclipse the true genetic diversity of same strain sequences isolated from a single epidemic leading to erroneous phylogenetic interpretations. Results from this study establishes parameters to avoid such misinterpretations. Firstly, the comparative accuracy (against Illumina generated consensus sequences) will drop below 99% without an adequate coverage of reads per base position, which can be unreliable for phylogenetics if other sequences are closely related (from the same epidemic in same location, for instance). This may still be adequate for cross sero/genotype or cross-epidemic analysis of the same genotype, but this aspect was not tested in this study. Secondly a very conservative bootstrap cut-off of 99% with nanopore sequencing data was required to produce comparable results to short read sequencing in our pipeline. Using a more liberal bootstrap cut-off may increase the risk of false clustering. Thirdly, it is recommended to use the full genome instead of subsequences for phylogenetic analyses to identify all true clusters and avoid underestimation or overestimation of clustering. On one hand, the use of the full genome sequence is intuitive as it encompasses all genomic variation; on the other hand when using an error prone sequencing platform, errors also accumulate as the length of the sequence increases. However, as shown here by clustering of Illumina consensuses with their nanopore counterparts in the full combined phylogenetic tree, provided the other parameters of sequence coverage and tree building are met, near-full length nanopore sequences were reliable for accurate demonstration of phylogenetic relationships. As it is not always feasible to concurrently sequence with short read technology for validation, these results will be useful to researchers for judicious use of stand-alone nanopore data.

The in-house developed Nano-Q bioinformatics tool successfully differentiated within-host variants and demonstrated these to have less variation (as expected) than between host variants except on 3 occasions where variants from two subjects were mixed in a single cluster (6 out of 26) in the phylogenetic tree (Fig. 5). Although unlikely, this could be due to a donor-recipient transmission pair which cannot be ruled out without data on temporal and spatial proximity of the subjects. A more likely explanation is the insufficient number of subjects in the full phylogenetic tree. As more subjects (and hence variants) are included in the phylogenetic analysis, the distribution of patristic distances widens, and this may facilitate segregation of sub clusters from a previous large single cluster.

To our knowledge this is the first attempt to describe within host variants of dengue virus using high throughput sequencing. This opens the door to prospects of understanding within host and within vector adaptations of the virus, the strain switches that happen over time in the same location and the role of viral epistasis in driving these changes. If dengue vaccines become widely accepted, these methods will also help to identify mutation patterns of viruses in breakthrough infections or during in vitro simulations of potential viral escape against neutralizing antibodies.

### Limitations

The near full-length genome amplification assay described here has a low success rate for samples with low viral loads for which the traditional approach to sequencing may be more suited. However, it is not possible to reconstruct within-host variants when subgenomic regions are amplified in separate PCRs. The final sample size for comparison with short read sequencing in this study was small (n-14) after filtering out non-DENV2 sequences and those with inadequate coverage (with either of the sequencing platforms). Most samples failed to meet minimal coverage requirement probably because size selection was combined with PCR barcoding as the standard approach which was recognized as inefficient during subsequent experiments. The samples were mostly of DENV2 serotype as this was the dominant serotype in Sri Lanka when the samples were collected (in 2018/19). However, the findings and interpretations are unlikely to change based on the serotype. DENV4 infected samples were few and all had a low viral load below the sensitivity of the assay. While it is assumed that the protocol should work regardless of this serotype it is noted that DENV 1, 2 and 3 are phylogenetically more different from DENV4. This was reflected in primer design where DENV4 required a different primer for each round of amplification and the suitability of this assay therefore needs to be subsequently confirmed for this serotype. Unlike the comparison of consensus sequences, there is no accuracy benchmark to compare within-host variants identified by the Nano-Q tool. The tool has been successfully tested for its ability to reproduce the constituent sequences (in the correct frequencies) in hepatitis C virus clone mixes which have a similar genomic length and a structure to dengue virus (manuscript in preparation). The same parameters calibrated for HCV were used for DENV as we do did not have access to cell cultures or clones of dengue virus to independently verify the results. There is however a risk of accepting a false variant as a true variant at very low frequencies of abundance. We do not recommend using an arbitrary cut-off of abundance (e.g. < 5% abundance) to neglect any variants below that threshold as this cut-off varies depending on depth of sequencing per sample. Instead users can utilize the safeguards provided in Nano-Q tool to improve the reliability of variants detected (e.g. adjusting base quality threshold for accepting a variant base, increasing minimum cluster size that are used to generate a mini-consensus for a variant, reducing the hamming distance threshold per cluster—see supplementary methods for details). It is recommended to perform a sensitivity analysis by adjusting these user defined variables and select variants that are consistently detected. The Nano-Q tool cannot remove PCR duplicates. For more accurate estimation of variant frequencies, these should ideally be removed from the input alignment to the tool. There may be two options in this regard: (a) direct RNA sequencing (unsuitable for samples with low viral loads),(b) using unique molecular identifiers (UMI) in primers to tag each amplicon during PCR to identify true duplicates (more suitable for the assay described in this paper). We did not use UMIs in the work described in this paper and hence it is possible that our frequency estimates of major within-host variants are biased towards the higher side due to the influence of PCR duplicates. We do not recommend retrospective removal of similar reads in an alignment without UMIs, on the assumption that they are “PCR duplicates” given the risk of removing true variant reads; This may bias the frequency estimates of major variants towards the lower side.

In conclusion, this study demonstrates the potential of a novel assay and analytic pipeline to characterize near full-length DENV amplicons using nanopore sequencing and to differentiate near full-length within-host variants. This study also establishes parameters for appropriate use of nanopore generated DENV consensuses when performing phylogenetic analyses of closely related sequences from the same epidemic (given the inherent error rate in nanopore sequencing) by comparing the results against state-of-the-art paired end short read (Illumina) sequencing. In contrast to existing methods, our assay provides a cost-effective solution for the accurate identification of phylogenetic relationships encompassing genetic variation across the entire genome, which will benefit low- and middle-income countries where dengue is endemic and increasing in incidence. Our work provides a tangible step forward to help establish real-time molecular surveillance strategies to monitor and predict changes in viral epidemics that have previously been restricted by the inaccessibility of sequencing technologies.

### Methods

The DENV near-full-length amplicon generation assay is inspired by a similar method previously developed for near-full-length pan-genotypic HCV sequence amplification by several investigators of this paper^20^. The protocol involves RNA extraction, reverse transcription of viral RNA to cDNA followed by two rounds of PCR to generate double stranded viral amplicons. The final product is purified (with or without size selection; please see below) and sequenced with ONT. The full protocol is detailed in the supplementary methods.

### Primer design

To ensure universal application of the assay to samples isolated worldwide, primers were designed against the conserved regions from alignments of serotype-specific dengue viruses isolated worldwide from 2005 to 2018. The sequences isolated from human hosts were downloaded from the Viral Pathogen Resource (ViPR) database^21^ and aligned with MUltiple Sequence Comparison by Log-Expectation (MUSCLE, Version 3.8.31) algorithm^22^. As Serotypes 1 and 2 had many sequences (Serotype 1: 1868, Serotype 2: 1411), only those sequenced from 2010 (Serotype 1: 507, Serotype 2: 475) were included to reflect recent viral evolution. Serotypes 3 and 4 had a smaller number of sequences (Serotype 3: 846, Serotype 4: 216), and the threshold was extended backwards to 2005 as the sequence availability and diversity was insufficient for entries after 2010 (Serotype 3: 491, Serotype 4: 158) as sequences were biased towards a few large studies from the same geographical region. Each alignment was used to generate a neighbor joining tree with 100 bootstrap replicates and clusters with at least 70% of bootstrap support. A representative sequence from each cluster was randomly extracted to a second serotype specific alignment. The list of extracted sequences (accession numbers), the country of origin and year of isolation are shown in Supplementary Tables 4, 5, 6 and 7. This alignment served as a reference (unbiased by large datasets with similar sequences from the same epidemic) for primer design. Conserved areas were identified within the 3′- and 5′ UTR from these alignments and serotype specific primers were designed using the Geneious Prime 2019.2.3 (https://www.geneious.com) software. The primer list is shown in Table 2 and Supplementary Methods.

**Table 2:** Primers for the full-genome amplification of dengue virus.

Region*	Round	Sense	Serotype	Sequence (5′-3′)
3′UTR	RT	−	DENV-1,2,3	CATTTTCTGGCGTTCTGTGC
		−	DENV-4	TGGTCTTTCCCAGCGTCAAT
5′UTR-NS5	PCR Outer	+	DENV-1,2,3,4	AGTTGTTAGTCTRYGTGGAC
		−	DENV-1,2,3	CATTTTCTGGCGTTCTGTGC
		−	DENV-4	TGGTCTTTCCCAGCGTCAAT
5′UTR/Capsid—NS5	PCR Inner	+	DENV-1	GTTTCGAATCGGAAGCTTGC
		+	DENV-2,4	CTGAAACGCGAGAGAAACCG
		+	DENV-2**	AGAGAAACCGCGTGTCRACT
		+	DENV-3	TGGATCACAGTTGGCGAAGA
		−	DENV-1,2,3	TCTGTGCCTGGAATGATGCT
		−	DENV-4	GGGTCTCCTCTAACCGCTAG

*Start and end of each amplicon according to positioning of nested PCR primers for each serotype are as follows; DENV1 (Reference: KM204119)—32 (5′-UTR) to 10,696 (3′-UTR), DENV2 (Reference: KM204118)—142 (Capsid) to 10,683 (3′-UTR), DENV3 (Reference: KU050695)—169 (Capsid) to 10,656 (3′-UTR), DENV4 (Reference: KR011349)—144 (Capsid) to 10,453 (3′-UTR).

**Can use either one of the primers for DENV2.

### Clinical samples

The clinical samples used to test the protocol were obtained in two batches. For optimization of the assay, DENV1 and 2 samples were imported from Singapore while a different set of samples representing all serotypes were imported from Sri Lanka for implementing the optimized assay. The second set of samples originate from the ongoing Colombo Dengue Study^23^ which recruits clinically suspected dengue patients within the first 3 days of fever. Dengue infection was confirmed by an NS1 antigen test or a quantitative PCR (qPCR) which amplified a small segment of the genome as described previously^24^, enabling serotyping of the sample. Once the diagnosis was confirmed, a 1 ml frozen serum aliquot was transferred in dry ice to The Kirby Institute, UNSW Sydney, Australia for all subsequent analyses described in this paper.

### Ethics statement

Ethics approval for work described in this paper has been granted from University of New South Wales (HC180015). All patients were above 18 years old and were recruited following written informed consent. The study was carried out according to all relevant ethical regulations.

### RNA extraction and amplicon generation

RNA extraction from thawed serum samples was performed using the QIAmp viral RNA mini kit (Cat. No: 52906, QIAGEN, Australia) following the manufacturer’s instructions with minor modifications as described in the supplementary methods. Reverse transcription of viral RNA was carried out using SuperScript III First-Strand Synthesis System (Cat. No: 18080051, Life Technologies, Australia) and a serotype specific 3′-UTR primer (DENV lacks a poly-A tail). The nested PCR was completed with serotype specific DENV primers using Takara LA taq DNA polymerase (Catalogue no: RR002B, Scientifix life, Australia). There was a significant conservation of the primers across serotypes 1,2 and 3 which allowed a common primer to be used for all serotypes in specific steps of the protocol (Table 2). The final products were size selected by gel extraction, picking the band at 10 kb (Monarch DNA Gel Extraction Kit, Cat no: T1020S, New England Biolabs, USA) and the elute was purified with magnetic beads (Agencourt AMPure XP, Beckman Coulter, USA, Cat: A63881) according to manufacturer’s instructions.

### Sequencing

ONT sequencing was carried out according to manufacturer’s protocols by a licensed external service provider (The Kinghorn Centre for Clinical Genomics, Sydney) on a GridION sequencer using R9.4 flow cells using the ligation sequencing kit (SQK-LKS109). Barcoding of PCR products was carried out with either (a) ligation barcoding (EXP-NBD104 and/or EXP-NBD114) or (b) PCR barcoding (SQK-PBK004) expansion pack. In the latter method, adapters were incorporated to the PCR product during the final round of the nested PCR, and these were used to attach a nanopore specific barcode in an additional round of PCR. PCR barcoded products were size selected and purified for a second time after the PCR to attach the barcodes. Ligation barcoding can pool up to 24 samples with the latest kits while PCR based barcoding can pool up to 96 samples for sequencing in a single ONT flow cell. However, the ligation barcoding kits available at the time of experiments could only handle 12 samples at a time. Signal-level data from the sequencer was base-called, filtered for high-quality reads (mean Q-score > 7) and demultiplexed with Guppy (version 2.3.5 and 3.0.3, https://staff.aist.go.jp/yutaka.ueno/guppy/) and further processed with Nanopolish algorithm (version 0.11.1, https://github.com/jts/nanopolish).

To compare the accuracy of ONT reads, a subset of amplicons was sequenced with paired-end short read sequencing (Illumina, NextSeq 500 HO 2 × 150 bp flowcell with Nextera XT library preparation kit). The cost per sample (pre-sequence library preparation and sequencing without service charges) were calculated and compared between the two platforms.

### Evaluating the impact of size selection

An experiment was carried out to assess the difference of size selection vs. non-size selection, on the number of final full-length nanopore sequences per sample (size selection was nonetheless considered as the default option for processed samples). Four DENV2 samples were amplified in two batches under the same experimental conditions and one batch was submitted for ONT sequencing with PCR barcoding without any size selection while the other batch was size selected prior to PCR barcoding and sequencing. Processed full-length reads were compared across the two batches.

### Consensus sequence generation and phylogenetics

Each demultiplexed patient-specific nanopore read was aligned against four random serotype-specific full length DENV genomes (as references) using the Minimap2 aligner (version 2.17)^25^ to produce an alignment. Now it is possible to implement this as a plugin within the Geneious Prime 2020.0.5, (https://www.geneious.com) platform to generate an alignment and a consensus sequence without using a command line. Alternatively, a binary alignment generated on a command line execution can be sorted and indexed with samtools (version 0.1.19)^26^. The consensus from these alignments were used as a custom reference sequence for a second round of aligning to minimize any bias in the original reference sequence. The same original reference sequence was used to align Illumina reads using the Bowtie 2 aligner (implemented in Geneious Prime 2020.0.5, https://www.geneious.com) as described by the authors previously for the Hepatitis C virus^19^. The majority base at each position in the alignment was incorporated to the consensus sequence by visualizing the alignments in Geneious Prime app. The consensuses generated by each sequencing method (nanopore vs. Illumina) per subject were manually inspected and aligned against each other for pairwise comparisons.

Sequences from the same epidemic can have little intrinsic variation and this may be eclipsed by the inherent error rate of nanopore sequencing leading to erroneous interpretations in phylogenetics. To explore this further, Maximum likelihood phylogenetic trees were built with the RAxML tool^27^ (1000 bootstrap replicates, 10% burn-in when generating the consensus tree) using DENV consensus sequences generated from both nanopore and paired-end short read (Illumina) platforms. This analysis assumed the state-of-art Illumina technology to be the gold standard and was restricted to DENV2 genomes as the number of sequences from other serotypes were too small for a meaningful phylogenetic analysis.

### Within-host variant identification

All sequenced samples were used as proof of concept to demonstrate the capacity of nanopore reads to differentiate within host variants without the need for haplotype reconstruction using a bioinformatics pipeline (Nano-Q tool) developed by authors to differentiate within host variants and their relative abundance from nanopore data (Supplementary Methods). This tool uses a sorted and indexed .bam file of sequence data as the input (aligned with Minimap2) and implements a hierarchical clustering algorithm combined with phylogenetics to identify read clusters with a close hamming distance and generates a consensus sequence for each cluster. The tool was initially developed and calibrated for the hepatitis C virus but given the structural similarities it can also be used for DENV. A full description of the Nano-Q tool and its development is published elsewhere (manuscript submitted), and the source code is publicly available at https://github.com/PrestonLeung/Nano-Q. The input parameters used for estimating DENV within host variants and safeguards against detecting false variants are detailed in Supplementary Methods.

## Supplementary information

Supplementary Information 1.

Supplementary Information 2.

Supplementary Information 3.

Supplementary Information 4.

Supplementary Information 5.

Supplementary Information 6.

Supplementary Information 7.

## Accession numbers

All 51 DENV sequences described in this paper have been deposited in Genbank under the following accession numbers; MT006136–MT006186.
