# Supplementary material for: Single molecule, near full-length genome sequencing of dengue virus
Source: Sci Rep. 2020 Oct 23;10:18196. doi: 10.1038/s41598-020-75374-1 (PMC7584602; doi:10.1038/s41598-020-75374-1)
Supplement: Supplementary file 1 — Supplementary Information 1. [file 41598_2020_75374_MOESM1_ESM.pdf]

Supplementary material

Single molecule, whole genome sequencing of dengue virus

Thiruni N. Adikari, Nasir Riaz, Chathurani Sigera, Preston Leung, Braulio M Valencia, Kirston Barton, Martin A. Smith, Rowena A. Bull, Hui Li, Fabio Luciani, Praveen Weeratunga, Tun-Linn Thein, Vanessa W.X. Lim, Yee-Sin Leo, Senaka Rajapakse, Katja Fink, Andrew R. Lloyd, Deepika Fernando, Chaturaka Rodrigo

Supplementary methods

RNA extraction, reverse transcription and PCR amplification

<u>RNA extraction</u>

RNA extraction was done with the QIAamp viral mini RNA kit (catalogue no. 52906) according to manufacturers instructions with the following modifications; a) 280µl of plasma/serum was used instead of 140µl and the volumes of regents up to the washing steps was doubled, b) During the preparation of lysis buffer, instead of the carrier RNA provided by the manufacturer, 2µl of yeast RNA (Ambion, AM7118) was used per 280µl of sample, c) mixing was done gently and vortexing was avoided altogether, d) the final elution volume was kept at 40-50µl and elution was completed with RNA storage solution (Ambion, AM7000) instead of the elution buffer provided by the manufacturer.

Primers

Region	Round	Sense	Serotype	Primer name	Sequence (5'-3')
3'UTR	RT	-	DENV-1,2,3	D1R1	CATTTTCTGGCGTTCTGTGC
		-	DENV-4	D4R1	TGGTCTTTCCCAGCGTCAAT
5'UTR-3'-UTR	Outer	+	DENV-1,2,3,4	d1s1C	AGTTGTTAGTCTRYGTGGAC
		-	DENV-1,2,3	D1R1	CATTTTCTGGCGTTCTGTGC
		-	DENV-4	D4R1	TGGTCTTTCCCAGCGTCAAT
5'UTR/ Capsid-3'-UTR	Inner	+	DENV-1	D1F2	GTTTCGAATCGGAAGCTTGC
		+	DENV-2,4	D2F2	CTGAAACGCGAGAGAAACCG
		+	DENV-2*	D2F2_2	AGAGAAACCGCGTGTCRACT
		+	DENV-3	D3F2	TGGATCACAGTTGGCGAAGA
		-	DENV-1,2,3	D1R2	TCTGTGCCTGGAATGATGCT
		-	DENV-4	D4R2	GGGTCTCCTCTAACCGCTAG

**an alternative if D2F2 does not work*

<u>Reverse transcription (RT)</u>

RT is performed with Superscript III First –Strand Synthesis System for RT-PCR. Invitrogen, Cat no. 18080-051.

RT reaction mix

**Reagent**	**Quantity/20 µl rxn.(µl)**	**Final Concentration**
10x RT Buffer	2	1x
$MgCl_2$ (120 mM)	1	6 mM
Betaine (5 M)	4	1 M
DTT (100 mM)	2	10 mM
RNaseOUT	1	-
Superscript III RT	1	9U

1) Make up enough RT reaction mix for the number of reactions plus one. Make up reaction mix on ice. Do not add template.
2) Pipette 1 µl of Primer (see above, 10 µM) and 1 µl of dNTPs (10 mM) into each 200 µl PCR tube.
3) Add 7 µl of extracted RNA to the tubes containing primer and dNTPs. (Do not add in pre-PCR room) and incubate at 65°C for 5 mins and then place on ice for 1 min.
4) Add 11 µl of RT reaction mix to RNA/primer mix on ice and cycle as below.

RT cycling conditions: 49°C for 65 min, 85°C for 5 min, add 1 µl of RNase H and incubate for 37°C for 20 mins. Hold at 12°C, Proceed to PCR immediately.

<u>Polymerase Chain Reaction</u>

PCR performed with Takara LA Taq. (clontech cat no. RR002M).

1) Make up enough PCR reaction mix for the number of reactions plus one. Do not add template.

2) Add 45µl of PCR reaction mix to each PCR tube.

3) Add 5µl of template RNA to each tube (ie. DNA from above)

PCR reaction mix

Reagent	Quantity/50 µl rxn. (µl)	Final Concentration
10x Buffer with Mg	5	1 x
dNTP (2.5mM)	8	0.4mM
Primer F (10 pmol/µl)	1	0.2 pmol/µl
Primer R (10 pmol/µl)	1	0.2 pmol/µl
dW	29.5	-
Taq enzyme mix (5 units/µl)	0.5	1 x
cDNA	5	-

PCR cycling conditions:

94°C for 1 min

PCR step, 10 cycles at:

Denaturation at 94°C, 30 secs

Annealing at 55°C for 20 secs

Extension at 68°C for 10 min

PCR step, 20-25 cycles at:

Denaturation at 94°C, 30 secs

Annealing at 57°C for 20 secs

Extension at 68°C for 10 min (+20 sec/cycle)

Final extension 68°C for 5 min then hold at 4°C.

<u>Nested Polymerase Chain Reaction</u>

1) Make up enough PCR reaction mix for the number of reactions plus one. Do not add template.
2) Add 45µl of PCR reaction mix to each PCR tube.
3) Add 5µl of template DNA to each tube (ie. from the step above)

Nested PCR reaction mix

Reagent	Quantity/50 µl rxn. (µl)	Final Concentration
10x Buffer with Mg	5	1 x
dNTP (2.5mM)	8	0.4mM
Primer F (10 pmol/µl)	1	0.2 pmol/µl
Primer R (10 pmol/µl)	1	0.2 pmol/µl
dW	29.5	-
Taq enzyme mix (5 units/µl)	0.5	1 x
1st round product	5	-

PCR Cycling conditions:

94°C for 1 min

PCR step, 10 cycles at:

Denaturation at 94°C, 30 secs

Annealing at 58°C for 20 secs

Extension at 68°C for 9 min

PCR step, 20-25 cycles at:

Denaturation at 94°C, 30 secs

Annealing at 60°C for 20 secs

Extension at 68°C for 9 min (+20 sec/cycle)

Final extension 68°C for 5 min then hold at 4°C.

Separate PCR products on a 0.8% agarose gel, stain with GelRed and visualise under UV light. Size select the 10kb band with gel extraction and purify with Agencourt Ampure XP magnetic beads (Beckman Coulter, A63881) prior to submitting for sequencing.

Nano-q tool for within host variant identification

Nano-q is currently executable on Linux systems, on command prompt. It uses contains nine user defined parameters and three optional parameters. This tool and its instructions for use are publicly available at: https://github.com/PrestonLeung/Nano-Q

Nano-q tool was executed for dengue samples with the following command

python nano-q.py -b ../<example.sorted.bam> -l 10000 -nr 1 -q 5 -j 50 -c 1 -ht 400 -mc 20

-l: length cut-off per read.

-nr: number of references for the alignment (usually one)

-q: threshold for base quality score for cleaning reads.

-c: starting codon (in the reference) for eligible reads.

-ht: Hamming distance cut-off where all reads within this value will fall into a single cluster.

-mc: minimum acceptable number of reads per cluster.

The values for each of these parameters were set after performing a sensitivity analysis on an in-silico data set of Hepatitis C virus plasmids (both between and within host) mixed in known proportions. These experiments, development and calibration of Nano-q tool will be published elsewhere.

Safeguards against detecting false variants

There are five safeguards in Nano-q tool to avoid detection of false minor variants

1. setting the -q parameter high. This will ensure the detected single nucleotide polymorphisms are of higher quality and hence reliable.

2. increasing the -mc parameter. When similar reads are arranged to clusters to generate a mini consensus, each cluster will have more reads for better reliability

3. decreasing -ht parameter. This will ensure that very similar reads are included in a cluster and together with a high -mc parameter, will further increase the reliability of a true cluster

4. decreasing -l parameter. If non-size selected amplicons are sequenced, reducing -l may ensure more reads are available for the analysis (more information to make a reliable estimate)

5. using optional -d parameter. The code has the option to plot a dendrogram (parameter -d) which shows the user how the hierarchical clustering is currently cutting the clusters. This feature provides support to determine what to set for parameter -ht.

For all above parameters, a default number is encoded into the algorithm, based on sensitivity analysis performed using in silico HCV sequence mixes, to act as a starting point. Being more conservative in setting these parameters will increase the reliability of major variants but may also lose true minor variants. A sensitivity analysis is highly recommended by varying each of the parameters when a new dataset is used with the tool.

Supplementary Figure 1

Maximum likelihood phylogenetic trees of DENV2 nanopore consensuses made from each of the subgenomic segments compared against that of the near full-length genome.

Caption: Capsid-PrM, envelope and NS5 region trees identify only one of the two true clusters while NS3 region identifies none. NS1, NS4 trees identifies both true clusters but another additional cluster. NS 2 region identifies only the two true clusters but cannot resolve relationships within the larger cluster as can be done with the near full-length genome tree.

Supplementary tables

Table S1. Cq values and estimated viral loads of all the samples included in the study

Sample	Serotype	Cq	PFU/mL	Full genome amplification
S1	DENV2	37.62	Not available	
S2	DENV2	28.76	Not available	
S3	DENV2	16.46	Not available	Successful
S4	DENV2	19.97	Not available	Successful
S5	DENV2	16.5	Not available	Successful
S6	DENV2	29.92	Not available	Successful
S7	DENV2	30.42	Not available	
S8	DENV2	22.23	Not available	Successful
S9	DENV4	38.36	Not available	
S10	DENV2	20.69	Not available	Successful
S12	DENV2	13.45	Not available	Successful
S13	DENV2	15.49	Not available	Successful
S14	DENV2	33.89	Not available	
S15	DENV2	23.4	Not available	Successful
S16	DENV4	36.67	Not available	
S17	DENV2	19.24	Not available	Successful
S18	DENV2	29.51	Not available	Successful
S19	DENV2	22.81	Not available	Successful
S20	DENV2	37.81	Not available	
S21	DENV2	19.93	Not available	
S22	DENV2	20.59	Not available	Successful
S23	DENV2	33.26	Not available	
S24	DENV2	22.91	Not available	Successful
S25	DENV2	26.28	Not available	Successful
S26	DENV4	40.52	Not available	
S28	DENV2	24.21	Not available	Successful
S29	DENV4	39.86	Not available	
S30	DENV2	19.17	Not available	Successful
S31	DENV2	36.43	Not available	
S32	DENV2	25.85	Not available	Successful
S33	DENV4	38.42	Not available	
S37	DENV2	25.5	Not available	Successful
S38	DENV2	24.46	Not available	Successful
S39	DENV2	25.06	Not available	
S41	DENV2	30.11	Not available	
S42	DENV2	22.74	Not available	
S45	DENV2	21.75	Not available	Successful

S46	DENV2	32.86	Not available	
S47	DENV2	26.98	Not available	
S48	DENV2	36.97	Not available	
S50	DENV3	39.51	Not available	
S53	DENV3	26.41	Not available	Successful
S54	DENV2	32.4	Not available	Successful
S56	DENV2	26.71	Not available	Successful
S65	DENV2	39	Not available	
S68	DENV2	30.33	Not available	
S69	DENV2	25.15	Not available	Successful
S71	DENV2	24.6	Not available	
S72	DENV2	26.19	Not available	Successful
S73	DENV2	20.94	Not available	Successful
S75	DENV1	27.27	Not available	Successful
S76	DENV1	35.43	Not available	Successful
S80	DENV2	30.98	Not available	
S81	DENV2	21.76	Not available	Successful
S82	DENV2	34.71	Not available	
S83	DENV2	40.05	Not available	
S86	DENV2	23.56	Not available	Successful
S91	DENV2	26.8	Not available	
S93	DENV2	22.68	Not available	
S94	DENV2	25.32	Not available	Successful
S96	DENV2	27.8	Not available	
S97	DENV2	32.37	Not available	
S99	DENV2	38.27	Not available	
S100	DENV2	32.37	Not available	
S101	DENV2	38.42	Not available	
S102	DENV2	35.46	Not available	
S103	DENV2	26.09	Not available	Successful
S106	DENV2	25.07	Not available	Successful
S107	DENV2	36.39	Not available	
S108	DENV2	35.15	Not available	
S109	DENV2	36	Not available	
S111	DENV2	21.9	Not available	Successful
S113	DENV2	24.26	Not available	Successful
S115	DENV2	28.98	Not available	
S117	DENV2	35.03	Not available	
S118	DENV2	39.12	Not available	
S124	DENV1	38.56	Not available	Successful
S125	DENV2	35.46	Not available	Successful
S128	DENV1	29.71	Not available	
S130	DENV2	24.7	Not available	Successful
S244	DENV2	20.09	19,876	

S245	DENV2	27.19	1400	
S252	DENV2	18.61	55,864	Successful
S255	DENV2	18.57	57,506	
S263	DENV2	19.91	57,506	Successful
S264	DENV2	20.21	18,372	Successful
S153	DENV1	32.29	175	Successful
S169	DENV3	24.48	215,005	Successful
S173	DENV1	27.22	6,825	Successful
S188	DENV1	27.97	3,952	Successful
S199	DENV3	27.58	3,531	Successful
S205	DENV1	25.79	2,035	
S228	DENV1	25.66	2,243	
S256	DENV1	20.09	2,243	
S263	DENV1	19.91	22,501	
S264	DENV1	20.21	18,372	
S279	DENV1	28.58	251	Successful
S303	DENV3	32.93	68	
S311	DENV1	27.73	476	
S312	DENV1	29.31	146	
S316	DENV1	32.61	12	
S331	DENV1	28.65	238	
S350	DENV1	25.68	309,098	Successful
S355	DENV1	25.68	261,627	Successful
S378	DENV3	31.51	7,333	
S379	DENV1	33.51	3,705	
S383	DENV3	23.11	1,860,288	Successful

Table S2. Viral loads (as plaque forming units per ml) corresponding to Cq ranges shown in figure 1*

Cq value range	Number of samples*	Mean (PFU/ml)**	95% Confidence limits
10 - 20	6	8,165,824	$2.71 \times 10^{3} – 2.45 \times 10^{10}$
20.01 - 25	46	1,119,438	$1.34 \times 10^{4} – 9.35 \times 10^{7}$
25.01 - 30	112	63,387	$6.08 \times 10^{2} – 6.61 \times 10^{6}$
30.01 - 40	135	2,780	$2.4 \times 10^{1} – 3.19 \times 10^{5}$

*This data is derived from 299 dengue samples tested with the same protocol, in the same machine by same operators. This includes samples where full-length genome was successfully extracted or failed for work described in this paper and other samples currently awaiting full-genome extraction. Please see next table for viral load for each sample described in this paper.

**Mean was calculated after log transformation and reconverted (inverse log) to give value in PFU/ml. The standard deviation (SD) and confidence limits (2 SD) was calculated as log numbers and upper and lower limits of the log distribution were reconverted as PFU/ml.

Table S3. Percentage pairwise identity of 33 consensus sequences generated by both nanopore and Illumina platforms (average genome length – 10.1 kb)

Sample No	Serotype	% identity between Illumina and nanopore sequencing
S3	DENV2	99.295
S5	DENV2	99.506
S8	DENV2	99.048
S12	DENV2	99.61
S13	DENV2	99.951
S15	DENV2	99.941
S17	DENV2	99.921
S18	DENV2	99.97
S19	DENV2	99.576
S22	DENV2	99.516
S24	DENV2	99.916
S25	DENV2	99.709
S28	DENV2	99.319
S30	DENV2	99.111
S32	DENV2	99.951
S37	DENV2	99.931
S45	DENV2	99.882
S54	DENV2	99.97
S56	DENV2	99.951
S69	DENV2	99.941
S73	DENV2	99.911
S75	DENV1	99.862
S86	DENV2	99.789
S94	DENV2	99.941
S111	DENV2	99.299
S252	DENV2	99.679
S264	DENV2	99.936

S153	DENV1	99.941
S173	DENV1	99.931
S188	DENV1	99.902
S199	DENV3	98.55
S279	DENV1	99.897
S169	DENV3	99.671
Average		99.7067879

ST 4 – 7. Genbank accession numbers of public database sequences used to design primers for the full-length dengue genome amplification assay

Table S4. DENV1 reference sequences for primer design

Accession number	Country of origin	Year of isolation
JQ048541	China	2011
KJ806961	Singapore	2014
KX452067	Malaysia	2014
MF033257	Singapore	2016
MF033244	Singapore	2016
KC762647	Indonesia	2010
KU666942	Malaysia	2014
MF033230	Singapore	2015
KY057369	Indonesia	2012
KU509263	Thailand	2012
KX255489	China	2015
MF033254	China	2016
KT827369	China	2011
KJ755855	India	2013
KP398852o	Sri Lanka	2014
KY672932	China	2015
KT827371	China	2011
KJ649286	Saudi Arabia	2011
KU094071	China	2015
JQ915080	New Caledonia	2010
KX380797	Singapore	2012
LC128301	Philippines	2016
KY057366	Indonesia	2012
JN544411	Singapore	2011
MG877557	Gabon	2012
KC692514	Argentina	2010
KJ189366	Puerto Rico	2010
KU509252	Venezuela	2010
MH450312	Venezuela	2015
JQ675358	USA	2010
KJ189369	Mexico	2011
KF973458	Nicaragua	2012
KY829115	St. Barthelemy	2016
KP188543	Brazil	2012
KX618705	India	2014
KU509255	India	2011
MF033255	Singapore	2016
KY921903	Singapore	2015

Table S5. DENV2 reference sequences for primer design

Accession number	Country of origin	Year of isolation
JX286518	Brazil	2010
KC294223	Peru	2010
KU509267	Guatemala	2010
KY474312	Ecuador	2014
MH215277	Venezuela	2016
KU094070	China	2015
KY672955	China	2015
LC410185	Thailand	2016
KX274130	Australia	2015
KJ010185	Pakistan	2011
JQ955624	India	2011
MH822945	India	2012
KY937188	China	2015
KY672953	China	2015
MH822940	India	2014
MK578533	China	2018
KY427084	India	2010
MG560143	India	2014
KY627763	Burkina Faso	2016
KU509274	Philippines	2010
KC131142	China	2012
MG779203	Kenya	2017
MF314189	Singapore	2016
KX452025	Malaysia	2014
MH010629	China	2017
MH823208	Indonesia	2014
KY921904	Singapore	2014
MK564486	China	2018
KX225485	China	2015
KC762678	Indonesia	2010
KX380813	Singapore	2012

Table S6. DENV3 reference sequences for primer design

Accession number	Country of origin	Year of isolation
JF295012	Cambodia	2007
EU482457	Vietnam	2006
KU509284	Thailand	2008
KJ622195	China	2013
MG721059	India	2016
JN662391	China	2009
FJ898441	Mexico	2006
FJ182041	USA	2005
FJ639817	Venezuela	2006
GQ868578	Colombia	2007
JF808121	Brazil	2007
MH888333	Bolivia	2011
FJ850089	Brazil	2006
FJ898474	Venezuela	2007
KJ189287	Peru	2008
EU854292	Venezuela	2005
KF973484	Nicaragua	2011
JN697379	Brazil	2006
KU509279	Philippines	2008
MF004386	Malaysia	2012
KY863456	Indonesia	2016
KY794786	Papua New Guinea	2010
GU370052	Singapore	2009

Table S7. DENV4 reference sequences for primer design

Accession number	Country of origin	Year of isolation
GQ398256	Singapore	2005
MK614089	China	2018
MH823210	Indonesia	2014
JX024758	Singapore	2010
LC069810	Japan	2015
JF741967	China	2010
KC762694	Indonesia	2007
KU509288	Indonesia	2010
KY921910	Singapore	2016
KC762695	Indonesia	2007
JQ915086	New Caledonia	2009
LC410197	Thailand	2016
KX224312	Singapore	2014
JN638572	Cambodia	2008
JQ513345	Brazil	2011
KP792537	Singapore	2011
MG272273	India	2016
KF041260	Pakistan	2009
KM190936	Thailand	2006
JQ513334	Brazil	2010
KY474335	Ecuador	2014
FJ882586	Venezuela	2007
JQ513331	Brazil	2010
JQ513342	Brazil	2011
KJ596666	Brazil	2012
MK514144	Haiti	2015

Supplementary data (Please see attached folder)

Datafile 1. Consensus full genome sequences of all 51 DENV sequences from this study (also deposited in Genbank – accession numbers MT006136 – MT006186)

Datafile 2. Alignment of 14 DENV2 sequences with a coverage > 100, sequenced with nanopore technology

Datafile 3. Same subjects as in datafile 2, sequenced with Illumina technology (sequences denoted with a "_I")

Datafile 4. Alignment of within host variants of DENV2. (Subject name is followed by frequency of the variant. Only unique variants are shown)

Datafile 5. Alignment of within host variants of DENV1 (Subject name is followed by frequency of the variant. Only unique variants are shown)
