# Supplementary figures and images for: Single molecule, near full-length genome sequencing of dengue virus
Source: Sci Rep. 2020 Oct 23;10:18196. doi: 10.1038/s41598-020-75374-1 (PMC7584602; doi:10.1038/s41598-020-75374-1)

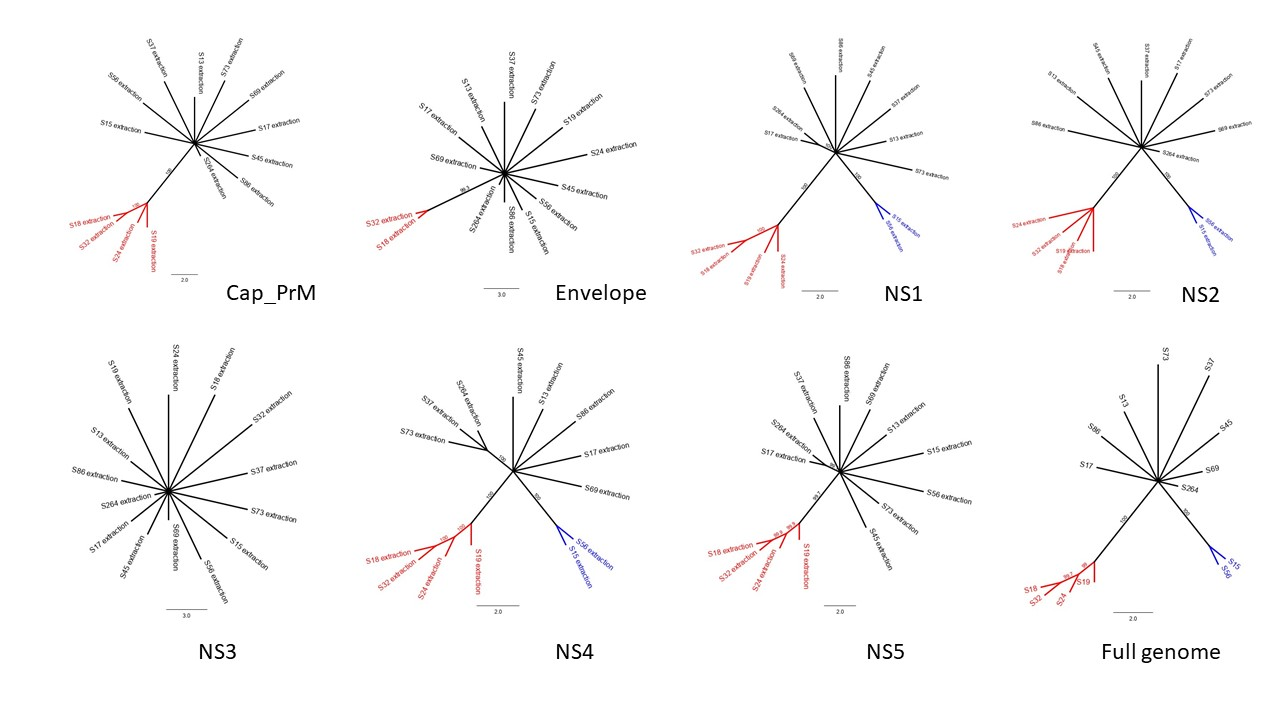

Supplement: Supplementary file 2 — Supplementary Information 2. [file 41598_2020_75374_MOESM2_ESM.tif]
